# Liver Impairment and Elevated Aminotransferase Levels Predict Severe Dengue in Vietnamese Children

**DOI:** 10.7759/cureus.47606

**Published:** 2023-10-24

**Authors:** Rang N Nguyen, Hue T Lam, Hung V Phan

**Affiliations:** 1 Pediatrics, Can Tho University of Medicine and Pharmacy, Can Tho, VNM

**Keywords:** dengue hepatitis, liver impairment, liver aminotransferase, severe dengue, non-severe dengue

## Abstract

Background: The degree of liver impairment in children with dengue infection varies from mild to severe injury. Aminotransferase levels may be useful in predicting severe dengue. This study aimed to evaluate the degree of liver impairment and determine whether elevated aminotransferases could be used to discriminate between non-severe and severe dengue in Vietnamese children.

Methods: This was a prospective cohort study of pediatric patients with confirmed dengue infection who were admitted to Can Tho Children's Hospital, Vietnam. The receiver operating characteristic (ROC) curve was used to discriminate the power of Aspartate transaminase (AST) or Alanine transaminase (ALT) to predict severe dengue.

Results: Two hundred and thirty confirmed dengue patients were enrolled, including 70% (161) patients with non-severe dengue and 30% (69) with severe dengue. This study indicates that 73.9% of patients had abnormal AST (＞40 U/L), and 34.8% of patients had abnormal ALT (＞40 U/L). The incidence of dengue patients with hepatitis (AST or ALT ≥ 4×ULN) and severe hepatitis (AST or ALT ≥ 10×ULN) were 18.7% and 17.0%, respectively. At a cut-off point of 120 U/L, AST's AUROC, sensitivity, and specificity were 0.93 (95% CI: 0.90-0.96), 82.5%, and 87.3%, respectively. At a cut-off point of 80 U/L, ALT's AUROC, sensitivity, and specificity were 0.89 (95% CI: 0.84-0,93), 87.5%, and 85.2%, respectively, for predicting severe dengue.

Conclusion: Elevated aminotransferase levels were associated with severe dengue, and AST/ALT were good markers for predicting severe dengue in Vietnamese children.

## Introduction

Dengue is a viral infection transmitted by Aedes mosquitoes. Dengue is an RNA virus that belongs to the genus Flavivirus and has four serotypes of dengue virus (DENV-1, 2, 3, and 4) [1]. It is a global health problem with an estimated 2.5 billion people at risk, mainly in countries in South and Southeast Asia, the Caribbean, Central, and South America, and recently in Africa [2]. In Vietnam, dengue morbidity per 100,000 population increased from 120 in 2009 (105,370 cases) to 194 in 2017 (184,000 cases); especially in 2019, Vietnam reported 320,000 dengue cases [3].

Dengue causes a wide spectrum of diseases, ranging from asymptomatic infection to a self-limiting febrile illness to severe dengue, a life-threatening condition characterized by increased capillary permeability and shock [4]. The degree of liver impairment in children with dengue infection varies from mild injury with an elevation of liver transaminases to severe injury with jaundice and liver failure [5-7]. The incidence of hepatic impairment is higher in dengue shock syndrome and dengue hemorrhagic fever [8-10]. A rise in aminotransferase levels can predict hepatic impairment. In a meta-analysis including 15 studies, Wang et al. reported that approximately 75-80% of dengue patients had abnormal aspartate aminotransferase (AST) and approximately 52-54% had abnormal alanine aminotransferase (ALT) [11]. Moreover, an elevated AST and ALT level is associated with the severity of dengue infections [12-15].

In the study of dengue in Malaysian adults, Sani et al. found that the composite index AST^2/ALT had a good parameter in identifying severe dengue [16]. Among children with dengue, Srivastava et al. observed an increase in transaminases with severity and that AST and ALT could be used to predict severe dengue [17]. 

This study aims to assess the severity of liver impairment in dengue patients and whether an increase in AST or ALT is useful for discriminating between mild and severe dengue in Vietnamese children.

## Materials and methods

Study design and setting

This prospective cohort study included children admitted to Can Tho Children's Hospital, Vietnam, with confirmed dengue infection. Sample recruitment was conducted from April 2018 to May 2020. All children between the ages of 1 and 15 who meet the WHO 2009 criteria for suspected dengue infection are eligible. Patients with a history of liver disease or hepatitis B or C were excluded from the study. Confirmation for dengue infection by Dengue NS1 Ag Rapid Test (SD Bioline) and/or Enzyme-linked immunoassay (ELISA) for dengue virus IgM.

Sample size

With a sensitivity value of 0.90, the margin of error of an estimate is 0.10, the number of objects required for sensitivity is 180, and the number required for specificity is 45; the total sample size required will be 225.

Procedures and data collection

The study included patients continuously until sufficient sample size was reached. The data were collected using a structured data collection format and included information about demographics (age, sex), clinical signs and symptoms (fever, nausea, vomiting, abdominal pain, hepatomegaly), hematology (WBC, hematocrit, platelets), ultrasonography (ascites, pleural effusion), and liver impairment-related data (bilirubin, albumin, AST, ALT).

In all dengue patients, AST and ALT were measured immediately after admission and every morning from days 3 to 5 of the illness. The highest values of AST and ALT were recorded. Treatment protocols and blood tests were based on Vietnam's National Guidelines for Diagnosis and Treatment of Dengue Fever [18]. 

Definitions

Severe dengue is defined by the presence of plasma leakage and/or fluid accumulation leading to shock or respiratory distress; and/or severe bleeding; and/or severe organ involvement, defined as AST >1000 U/L and/or ALT >1000 U/L, serum creatinine ≥3 times above baseline, myocarditis, and/or encephalitis. The non-severe dengue group is divided into patients with and without warning signs [19].

Liver impairment is defined as an increase in the AST and/or ALT levels at least twice higher than the upper limit of normal (ULN). Hepatitis is defined as an ALT level at least four times the ULN, and severe hepatitis is defined as an ALT level at least 10 times higher than the ULN. The ULN of ALT and AST was defined as > 40 U/L [20-21].

Ethical issues

Parents signed informed consent forms upon first attending the NICU. Ethical approval has been provided by the Science and Technology Board of Can Tho Children's Hospital and the Ethics Committee of Can Tho University of Medicine and Pharmacy (No: 734-QD-DHYDCT) 

Statistical analysis

Data were analyzed using Statistical Package for Social Sciences (SPSS) version 22.0 software. Categorical variables were expressed as numbers and percentages, while continuous variables were expressed as median and interquartile ranges. Pearson's Chi-square test was used to compare categorical variables. Mann-Whiney tests were used for continuous variables with a non-normal distribution.

Univariate and multivariate logistic regression analyses were used to evaluate the odds ratios and 95% confidence intervals (CIs) of serum AST and ALT levels between non-severe and severe dengue patients after adjusting for other risk factors (age, sex, BMI, and albumin concentration).

The receiver operating characteristic curve method was used to discriminate the power of AST and ALT to predict dengue severity. Youden's J-statistic evaluated the optimal cut-off of AST and ALT to discriminate between severe and non-severe dengue. A P value of less than 0.05 was considered statistically significant.

## Results

Clinical features and laboratory characteristics in children with dengue fever

There were 336 suspected dengue patients admitted to Can Tho Children's Hospital, of which 230 confirmed dengue were included in this analysis. Male sex accounted for 53.9%. The median age of our children was 11 (IQR: 8-13) years old. Thirty percent (69/230) of the children had severe dengue, including shock (n=65), severe bleeding (n=2), and severe hepatitis with AST≥ 1000 U/L (n=2). There were 118 patients receiving oral paracetamol at a mean dosage of 41.4 ± 9.4 mg/kg per day (minimum 15.0 mg, maximum 68.5 mg/kg/24h). No death was recorded in this cohort. The clinical characteristics, ultrasonography, and laboratory investigations of children with non-severe dengue (NSD) and severe dengue (SD) are summarized in Table 1.

**Table 1:** Clinical features and laboratory investigations in children with non-severe and severe dengue AST: Aspartate AminoTransferase; ALT: Alanine AminoTransferase; APTT: Activated partial thromboplastin time; PT: Prothrombin time

	Total (n=230)	Non-severe dengue (n=161)	Severe dengue (n=69)	P value
Age (y)	11 (8-13)	11 (7-13)	11 (9-13)	0.188
Sex, male	124 (53.9)	91 (56.5)	33 (47.8)	0.225
Clinical features				
Fever	106 (46.1)	87 (54.0)	19 (27.5)	< 0.001
Nausea/vomiting	174 (75.7)	108 (67.1)	66 (95.7)	< 0.001
Abdominal pain	92 (40.0)	23 (14.3)	69 (100)	< 0.001
Hepatomegaly>2cm	98 (42.6)	30 (18.1)	68 (98.6)	< 0.001
Ascites	58 (25.2)	15 (9.3)	43 (62.9)	< 0.001
Pleural effusion	61 (26.5)	16 (9.9)	45 (65.2)	< 0.001
Hematology				
Hematocrit %	41.9 (38.9-45.8)	40.4 (38.1-43.5)	45.2 (42.0-49.7)	< 0.001
Leukocyte x10^9^/L	4.3 (3.0-5.9)	4.0 (2.9-5.5)	5.1 (3.8-6.7)	0.001
Platelets x 10^9^/L	60.0 (27.7-127.2)	100 (54-147)	25.0 (18.0-37.0)	< 0.001
Serum bilirubin(mg/dL)	0.32 (0.23-0.51)	0.28 (0.21-0.39)	0.54 (0.37-0.84)	< 0.001
Serum albumin (g/L)	36.8 (33.0-39.2)	39.1 (37.1-39.5)	29.1 (18.7-32.5)	< 0.001
APTT (sec)	34.6 (31.2-41.1)	31.3 (30.1-34.3)	46.3 (41.1-78.3)	< 0.001
PT (sec)	11.8 (11.2-12.5)	11.4 (11.0-12.0)	13.8 (12.6-15.2)	< 0.001
AST (IU/L)	56.7 (39.2-119.2)	43.3 (37.5-58.2)	146.0 (114.0-250.5)	< 0.001
ALT (IU/L)	30.1 (20.2-58.4)	23.0 (19.1-30.2)	86.0 (56.8-144.0)	< 0.001

The median levels of AST and ALT were significantly higher in SD patients than in NSD patients, 146.0 versus 43.3 U/L (P < 0.001) and 86.0 versus 23.0 U/L (P < 0.001), respectively (Table 1).

Liver impairment in children with severe and non-severe dengue

A total of 98 (42.6%) dengue fever patients had hepatomegaly. Patients with hepatomegaly had approximately three-fold higher liver transaminases. Hepatomegaly patients had a median and interquartile range (IQR) of 121 (76-196) and 63 (32-104) for AST/ALT, compared with 42 (37-56) and 23(19-30) for those without hepatomegaly (Mann-Whitney test: p<0.001)

A total of 170 (73.9%) patients had abnormal AST (>40 U/L), and 80 (34.8%) patients had abnormal ALT (>40 U/L). Of these, 18.7% of dengue patients had hepatitis (AST or ALT ≥ 4×ULN), and 17.0% had severe hepatitis (AST or ALT ≥ 10×ULN). AST levels greater than 1000 U/L were observed in two patients without acute liver failure. Patients with SD were more likely to develop hepatitis than those with NSD (39.1% vs.. 9.9%; P <0.001). Additionally, the incidence of severe hepatitis was higher among SD patients as compared to NSD patients (44.9% vs. 5.0%; P <0.001) (Table 2).

**Table 2:** Liver impairment in children with non-severe and severe dengue ULN: upper limit of normal AST: Aspartate AminoTransferase; ALT: Alanine AminoTransferase

Liver function test	Categories	Total (n=230)	Non-severe dengue (n=161)(%)	Severe dengue (n=69)(%)	P value
AST	None	60 (26.1)	60 (37.3)	00 (0.0)	<0.001
	Liver impairment	88 (38.3)	77 (47.8)	11 (15.9)	<0.001
	Hepatitis (x4 ULN)	45 (19.6)	17 (10.6)	28 (40.6)	<0.001
	Severe hepatitis (x10 ULN)	37 (16.1)	07 (4.3)	30 (43.5)	<0.001
ALT	None	150 (65.2)	136 (65.2)	14 (20.3)	<0.001
	Liver impairment	32 (13.9)	19 (11.8)	13 (18.8)	0.160
	Hepatitis (x4 ULN)	29 (12.6)	03 (1.9)	26 (37.7)	<0.001
	Severe hepatitis (x10 ULN)	19 (8.3)	03 (1.9)	16 (23.2)	<0.001
AST or ALT	None	60 (26.1)	60 (37.3)	00 (0.0)	<0.001
	Liver impairment	88 (38.3)	77 (47.8)	11 (15.9)	<0.001
	Hepatitis (x4 ULN)	43 (18.7)	16 (9.9)	27 (39.1)	<0.001
	Severe hepatitis (x10 ULN)	39 (17.0)	8 (5.0)	31 (44.9)	<0.001

Analysis of risk factors for severe dengue

Based on the univariate analysis, table 3 shows that a high hematocrit, a high leukocyte, a low platelet count, a high serum bilirubin level, a low serum albumin level, a prolonged APTT, a prolonged PT, and a high level of AST or ALT are associated with an increased risk of SD. After adjusting for other significant predictors of SD, high levels of AST and ALT were independent predictors of SD with aOR=33.2 (95% CI:1.63-678) and aOR=30.7 (95% CI: 1.51-623), respectively.

**Table 3:** Univariate and multivariate logistic regression analyses of risk factors for severe dengue OR: Odds ratio; aOR: adjusted OR AST: Aspartate AminoTransferase; ALT: Alanine AminoTransferase APTT: Activated partial thromboplastin time; PT: Prothrombin time

	*Univariate Analysis*		*Multivariate Analysis* * with AST included*		*Multivariate Analysis * *with ALT included*	
*Variables*	*OR (95%CI)*	P value	*aOR (95%CI)*	P value	*aOR (95%CI)*	P value
Hematocrit	1.24 (1.15-1.23)	<0.001	1.20 (0.88-1.64)	0.245	1.18 (0.87-1.61)	0.271
Leukocyte	1.21 (1.08 - 1.36)	<0.001	1.10 (0.69-1.75)	0.666	1.11 (0.71-1.75)	0.632
Platelets	0.94 (0.92 – 0.96	<0.001	1.01 (0.97-1.04)	0.559	1.01 (0.97-1.04)	0.611
Bilirubin	72 (14-351)	<0.001	1.05 (0.01-393)	0.821	1.36 (0.01-236)	0.906
Albumin	0.49 (0.40-0.60)	<0.001	0.56 (0.38-0.84)	0.005	0.57 (0.38-0.86)	0.008
APTT	1.47 (1.31-1.65)	<0.001	1.38 (0.98-1.94)	0.061	1.46 (1.06-2.00)	0.020
PT	4.98 (3.14-7.88)	<0.001	3.63 (0.81-16.2)	0.090	2.93 (0.77-11.1)	0.113
AST≥120U/L	1.03 (1.02-1.04)	<0.001	33.2 (1.63-678)	0.023		
ALT≥ 80 U/L	1.03(1.02-1.04)	<0.001			30.7 (1.51-623)	0.026

Discriminatory value of elevated aminotransferase levels for dengue severity in children

On admission, the area under the receiver operating characteristic curve (AUROC), the sensitivity, and the specificity of AST and ALT for their diagnostic value in identifying severe dengue were shown in Table 4.

**Table 4:** The AUROC, Sensitivity, and Specificity of AST and ALT AST: Aspartate AminoTransferase; ALT: Alanine AminoTransferase; AUROC: Area Under the Receiver Operating Characteristic Curve

	Optimal Cut-off	AUROC (95% CI)	Sensitivity	Specificity	P value
AST	120 IU/L	0.93 (0.90-0.96)	82.5%	87.3%	<0.001
ALT	80 IU/L	0.89 (0.84-0.93)	87.5%	85.2%	<0.001

**Figure 1:**
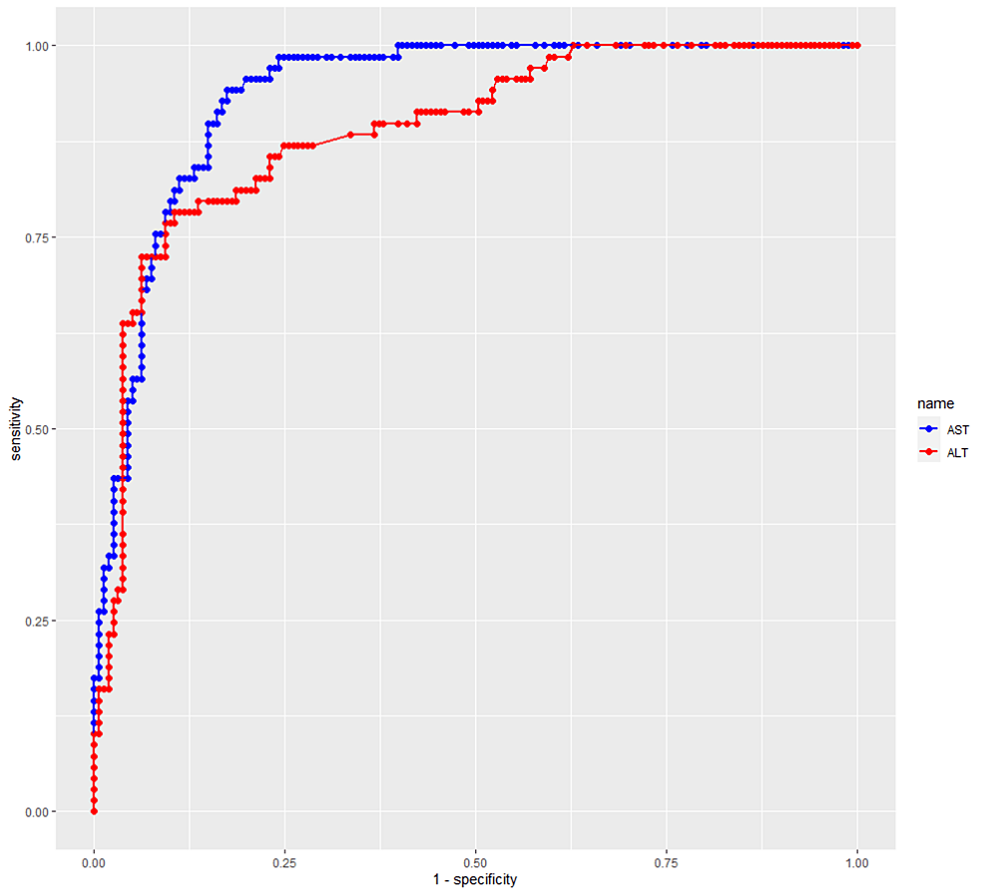
The Receiver Operating Characteristic Curve of AST and ALT in predicting of severe dengue. AST: Aspartate AminoTransferase; ALT: Alanine AminoTransferase

According to the ROC curve, an AST of 120 U/L had the best cut-off value (maximum Youden index value with 82.5% sensitivity and 87.3% specificity), and an ALT of 80 U/L had a sensitivity of 87.5% and a specificity of 85.2% for predicting SD (Figure 1).

## Discussion

The results of this study indicate that 73.9% of patients had abnormal AST (＞40 U/L), and 34.8% of patients had abnormal ALT (＞40 U/L). The incidence of abnormal liver enzymes in our study is similar to the results of a meta-analysis of 13 studies on abnormal liver enzymes in dengue patients, Wang and colleagues [11] found that 75% and 52% of patients with dengue fever had elevated AST and ALT, respectively, but lower than that of the previous study of 270 children with dengue that Kuo et al. found abnormal serum levels of AST and ALT in 93.8% and 82.2%, respectively [21]. In Vietnamese children with dengue hemorrhagic fever, abnormal levels of AST and ALT were observed in 97.7% and 37.3% of the patients, respectively [22]. This study found that AST levels were higher than ALT levels in dengue patients, similar to previous studies elsewhere [12-14]. This may be due to damage to organs other than the liver, such as the cardiac muscle, skeletal muscle, and kidney [23,24]

The level of transaminases and the incidence of hepatitis in our study was lower than in other studies, possibly because liver enzymes were measured during the early stage of the disease (3-5 days) when AST/ALT was not elevated to maximum levels around 9 days after the onset of fever [21]. Additionally, there are varying definitions of abnormal AST/ALT, ranging from 15 U/L to 88 U/L [11]

Liver injury is commonly seen in dengue fever to varying degrees. However, the exact mechanism of liver injury in dengue is unknown. There are several ways for this to happen: direct viral effects on hepatocytes and Kupffer cells, a T cell-mediated cytokine storm, decreased circulation that results in decreased hepatic perfusion, or drug hepatotoxicity [10, 25]. In this study, almost half of the patients received paracetamol but at a dose that did not cause liver toxicity.

In dengue infections, elevated ALT is a more specific indicator of liver damage than elevated AST. According to our study, 12.6% of dengue patients had hepatitis (ALT > 4 x ULN), of which 8.3% had severe hepatitis (ALT > 10 x ULN). This incidence of severe hepatitis was similar to that described in children with dengue in Thailand [26] but lower than among children with dengue in India. Jagadishkumar et al.reported that 17.27% of pediatric patients with dengue had severe hepatitis [27]. In our study, there were 2 patients with AST> 1000 U/L, and none developed fulminant hepatitis. In one study of Thai children, acute liver failure associated with dengue was reported to be 1.1% [28].

Almost all pediatric patients with dengue in Vietnam (97%) have elevated liver transaminases, especially AST [22]. However, AST/ALT cut-offs have not yet been studied for predicting severe dengue; in the WHO guidelines, AST/ ALT levels above 1,000 U/L are considered indicative of severe dengue; nevertheless. This value is rarely seen in severe dengue. Only two patients (2.8%) had AST/ALT levels over 1000U/I in our study. In predicting severe dengue in children, Srivastava G et al. [17] recommended a lower cut-off (635 U/L for AST and 376 U/L for ALT ). This cut-off had a high specificity (>98%) but a low sensitivity (5%). In our cohort, liver enzyme levels (AST/ALT) were not significantly elevated since they were measured in the early stage of the illness. As a result, we chose a low cut-off (120 U/L for AST and 80 U/L for ALT). This cut-off increases the sensitivity, but the specificity is not significantly reduced for predicting severe dengue. At a cut-off point of 120 U/L, AST's AUROC, sensitivity, and specificity were 0.93 (95% CI: 0.90-0.96), 82.5%, and 87.3%, respectively. At a cut-off point of 80 U/L, the AUROC, sensitivity, and specificity of ALT were 0.89 (95% CI: 0.84-0,93), 87.5%, and 85.2%, respectively, for the prediction of SD.

Compared to other studies on adults, our study had a more accurate prediction model for SD. A study by Lee et al. found that although the median AST and ALT levels of adults with SD were higher than those with NSD, liver aminotransferase levels could not discriminate SD from NSD. The AUROC of the AST in predicting SD was 0.62 (CI 95%:0.57-0.67) [29]. A study by Sani et al. on adults with dengue in Malaysia found that AST/ALT was useful in predicting SD, with an AUROC of 0.78 (95% CI: 0.68 - 0.89) and 0.69 (95% CI: 0.56 - 0.81), respectively. However, the composite index AST^2/ALT (AUC 0.83; 95% CI: 0.73 - 0.93) was the best-performing marker in identifying severe dengue on admission [16].

There are some limitations to our study. Firstly, The results of this study are not generalizable since it is a single-center study. Secondly, patients who had hepatitis B and C were excluded; however, other diseases (such as hepatitis A or typhoid) that may cause an elevated transaminase have not been ruled out. Lastly, dengue virus serotypes were not identified; however, most of the dengue virus serotypes prevalent in South Vietnam during that period were DEN-1 (37.8), DEN-2 (41.9%) and DEN-4 (18.8%) [1].

## Conclusions

Dengue is a major global health concern, especially in tropical and subtropical regions. In the present study, we found that most dengue children had abnormal liver enzymes, and a small number had severe hepatitis. In the first five days of illness, elevated levels of AST and ALT are good indicators of severe dengue, with optimal cut-off values of 120 and 80 U/l, respectively. Clinicians may use this AST/ALT cut-off to classify dengue severity in hospitalized children.
